# 

**Published:** 1883-06

**Authors:** 


### Pamphlets.

---

The Opieum Habit; its Successful Treatment by the Avena Satina.

A paper read before the New York State Medical Society. By. E. H. M. Sell, A.M., M.D.

Malignant Strictures of Pylorus and Intestines, with a brief review of the Historical Development of Modern Abdominal Surgery. By Reuben A. Nance, M.D., of Cleveland, Ohio.

Seventh Biennial Report of the Board of State Commissioners of Public Charities of the State of Illinois.

The Southern World.—We welcome this great family newspaper and journal to our exchange list. It is edited by Mr. W. P. Wooley, a rising young nan in the world of letters.

Among the brilliant contributions to its first number, we see the initial chapters of a serial from the pen of Col. B. F. Sawyer, of Atlanta, the De Sauseurs, a thrilling story from Southern history, whose romance is too touching and too true for bungling fiction. It will cause some peoples' ears to tingle and some a-lack-a-day saints will not believe it.

